# Development of a fully automated chemiluminescence immunoassay for urine monomeric laminin-γ2 as a promising diagnostic tool of non-muscle invasive bladder cancer

**DOI:** 10.1186/s40364-017-0109-4

**Published:** 2017-10-13

**Authors:** Masatoshi Nakagawa, Takashi Karashima, Masayuki Kamada, Eisaku Yoshida, Toru Yoshimura, Masanori Nojima, Keiji Inoue, Taro Shuin, Motoharu Seiki, Naohiko Koshikawa

**Affiliations:** 1 Diagnostic Division, Abbott Japan Co. Ltd., Chiba, Japan; 2 Division of Cancer Cell Research, Kanagawa Cancer Center Research Institute, 2-3-2 Nakao, Yokohama, 241–8515 Japan; 3 Department of Urology, Kochi Medical School, Kochi, Japan; 4 Center for Translational Research, Institute of Medical Science Hospital, University of Tokyo, Tokyo, Japan; 5 Division of Cancer Cell Research, Institute of Medical Science, University of Tokyo, Tokyo, Japan; 6 Faculty of Medicine, Institute of Medical, Pharmaceutical, and Health Sciences, Kanazawa University, Kanazawa, Japan

**Keywords:** Urine biomarker, Chemiluminescence immunoassay (CLIA), Monomeric laminin-γ2, Non-muscle invasive bladder cancer (NMIBC)

## Abstract

**Background:**

Monomeric laminin-γ2 in urine is a potential biomarker for bladder cancer. However, the current detection system uses an antibody that cannot discriminate between monomeric laminin-γ2 and the heterotrimeric γ2 chain of laminin-332, which may cause false-positive reactions. The present study aimed to develop a fully automated chemiluminescence immunoassay system using a specific monoclonal antibody against monomeric laminin-γ2.

**Methods:**

In total, 237 urine specimens (84 from patients with bladder cancer, 48 from patients with benign urological disease, and 105 from healthy donors) were collected, and monomeric laminin-γ2 values in the urine were measured using a fully automated chemiluminescence immunoassay.

**Results:**

The results revealed that laminin-γ2 values in patients with benign urological disease were comparable to those of healthy donors and that the chemiluminescence immunoassay’s lower limit of detection was 10 pg/mL (approximately 20-fold better than the sandwich enzyme-linked immunosorbent assay’s limit of 200 pg/mL). Moreover, the chemiluminescence immunoassay demonstrated that patients with bladder cancer, including non-muscle invasive bladder cancer (≤pT1), had higher laminin-γ2 values than patients with benign urological disease or healthy donors.

**Conclusions:**

These results suggest that urine monomeric laminin-γ2 may be a promising biomarker to diagnose cases of non-muscle invasive bladder cancer using a fully automated chemiluminescence immunoassay system.

**Electronic supplementary material:**

The online version of this article (10.1186/s40364-017-0109-4) contains supplementary material, which is available to authorized users.

## Background

The number of patients with bladder cancer (BC) has been increasing dramatically in developed countries, including Japan, as a result of the aging society [1, 2]. Early diagnosis and treatment of BC are needed to completely cure and control the disease; however, at present, there are no useful biomarkers to diagnose BC. Therefore, currently, urine cytology and cystoscopy are the major methods to diagnose BC. Although urine cytology can diagnose patients with high-grade muscle-invasive BC (MIBC) accurately, it is not suitable to diagnose low-grade non-muscle invasive BC (NMIBC), which accounts for approximately 70% of all BC cases. For example, because cancer cells are rarely released into the urine of patients with NMIBC (Ta, T1, or carcinoma in situ), urine cytology has a sensitivity of only 38–61% to identify NMIBC [3, 4]. Therefore, a specific biomarker is urgently required to diagnose NMIBC.

Laminin-γ2 (Ln-γ2) is a component of laminin-332 (Ln-332), which is mainly expressed in the normal epithelial basement membrane, and expression of monomeric Ln-γ2 (mono-Ln-γ2) alone is limited to the invasive front of malignant tumors [5–12]. Thus, mono-Ln-γ2 might be a useful biomarker for the invasion of malignant neoplasms.

In our recent study, we used western blotting and a sandwich enzyme-linked immunosorbent assay (ELISA) with conventional monoclonal antibodies (mAbs) recognizing Ln-γ2, and found that mono-Ln-γ2 accumulated in the urine of patients with BC [13]. In addition, patients with BC had higher Ln-γ2 values compared with patients with benign urological diseases; we even detected mono-Ln-γ2 in urine from patients with NMIBC (Ta, T1, or carcinoma in situ). Although nuclear matrix protein (NMP-22) and bladder tumor antigen (BTA) are approved currently as urine tumor markers to diagnose BC, their diagnostic accuracy is not sufficient for clinical diagnosis [2, 14, 15].

Our previous study revealed that urine Ln-γ2 testing provided greater accuracy to diagnose BC compared with NMP-22 or BTA, which suggested that Ln-γ2 might be a useful biomarker to diagnose BC [13]. In that study, we used a conventional mAb and polyclonal antibodies (pAbs) against Ln-γ2, and developed a sandwich ELISA with a lower detection limit of 200 pg/mL. However, some patients with benign disease had slightly elevated urine Ln-γ2 test results, [13] which suggests that our assay provided false-positive results, because Ln-332 is produced during basement membrane degradation in patients with inflammatory diseases of the urinary tract. If this assumption is correct, the false-positive results would limit our assay’s accuracy for the clinical diagnosis of BC. Therefore, the present study aimed to develop an improved system to detect urine Ln-γ2, using a specific mAb for mono-Ln-γ2 (2H2 mAb) and a fully automated chemiluminescence immunoassay (CLIA) [16, 17]. We evaluated the CLIA system using a large number of clinical specimens, and confirmed that mono-Ln-γ2 in urine is a potent biomarker to diagnose BC.

## Methods

### Anti-Ln-γ2 antibodies

A previous report has described a hybridoma cell that produces a monoclonal antibody (mAb) that targets monomeric Ln-γ2 (2H2) [16, 17]. The IgG1 protein was purified from the hybridoma serum-free culture medium using a Hi-Trap Protein G HP column (GE Healthcare). The epitope was an EGF-like 2nd loop (amino acids 82–138) in domain V of Ln-γ2 chain.

A rabbit polyclonal antibody (pAb) against a recombinant Ln-γ2 domain III (DIII) protein was produced in our laboratory. The DIII coding sequence (amino acids 383–608) was amplified from human Ln-γ2 cDNA (a gift from Professor Vito Quaranta, Vanderbilt University, Nashville, TN) using PCR. The PCR product was inserted into the pDEST15 expression vector (Thermo Fisher, Waltham, MA, USA) using pENTR/D-TOPO (Thermo-Fisher, Waltham, MA) and the Gateway system. The expression vector was then transformed into *Escherichia coli* BL21 cells, and the recombinant DIII protein was expressed as a GST-fusion protein. The recombinant DIII protein was purified using a GSTrap column (GE Healthcare, Little Chalfont, UK) and then used to immunize rabbits to produce the pAbs. A Hi-Trap Protein-G HP column was used to purify the pAbs against the DIII protein, and the pAb sample was further purified using a DIII protein-conjugated Sepharose column.

### Surface plasmon resonance (SPR) binding assay

The 2H2 or anti-Ln-α3 mAbs (R&D systems, Minneapolis, MN, USA) were immobilized on a carboxy-activated (CM5) sensor chip (GE Healthcare) using 1-ethyl-3-(3-dimethylaminopropyl)-carbodiimide/N-hydroxysuccinimide, and analyzed using a Biacore 3000 system (GE Healthcare). The binding assay was performed using the standard BIAcore HBS-EP buffer (GE Healthcare), and the purified mono-Ln-γ2 or Ln-332 proteins were diluted in HBS-EP buffer containing 0.3% (*w*/*v*) bovine serum albumin (BSA). The interactions between the proteins and mAbs were measured, and the binding data were analyzed using BIAevaluation Software. Regeneration was performed using a 1-min injection of 10 mM glycine-HCl (pH 2.0).

### Detection of Ln-γ2 or Ln-332 in urine using CLIA

The 2H2 or anti-Ln-α3 mAbs were immobilized on magnetic microparticles using carboxylic acid groups, and the pAbs to the DIII were labeled using acridinium. A two-step sandwich assay was performed for the fully automated CLIA using the ARCHITECT system (Abbott Laboratories, Chicago, IL, USA) (Additional file 1: Figure S1). Mono-Ln-γ2 and Ln-332 proteins were diluted in phosphate-buffered saline (PBS) containing 1% (*w*/*v*) BSA, 0.1% (*v*/v) Tween-20, and 0.1% (v/v) ProClin300, and were used to develop the assay standards. The standard concentrations of mono-Ln-γ2 were 0, 10, 20, 50, 100, 1000, 10,000, and 20,000 pg/mL. The standard concentrations of Ln-332 were 0, 100, 250, 500, 1000, and 10,000 pg/mL.

### Measurement of urine creatinine (CRE)

Urine creatinine values were measured using a colorimetric assay and the Determiner-L CRE system (Kyowa Medex, Tokyo Japan).

### Clinical urine specimens

Between July 2008 and November 2016, urine specimens were obtained from patients with BC, patients with benign urological diseases, and healthy donors (HDs) at the Department of Urology, Kochi Medical School Hospital. Specimens from HDs with no history of liver, kidney, or urological diseases were also collected at the Kochi Medical School Hospital (Table 1). All patients and HDs provided written informed consent, and the study protocol was approved by our institutional review boards (Kochi Medical School Hospital: 24–139; Institute of Medical Science, University of Tokyo: 20–52-0123; Kanagawa Cancer Center: Res-36). In total, 237 urine specimens were collected from 84 patients with BC, 48 patients with benign urological disease, and 105 HDs. All urine specimens were centrifuged for 5 min at 1000 rpm after collection from patients with BC, benign urological diseases and HDs and stored at −80 °C for 1–6 months and then, the specimens (250 μL) were subjected to CLIA after centrifugation for 10 min at 15 k rpm, 4 °C.

**Table 1:** Characteristic of the evaluated patients with bladder cancer, benign urological diseases and healthy donors

	No. of urine
Gender	
Male	169
Female	68
Bladder cancer	84
pTMN	
pT ≤1	44
pT ≥2	9
No information	31
Benign diseases	
BPH	26
Urinary Stone	5
Hematuria	4
OAB	3
Cyst	2
Others (NB, XGP)	8
Healthy donors	105

*BPH* benign prostate hyperplasia, *OAB* overactive bladder, *NB* neurogenic bladder, *XGP* Xanth pyelonephritis

### Statistical analysis

Analyse-it version 3.90.5 (Analyse-it Software, Leeds, UK) was used to generate all scatter plots, receiver operating characteristic (ROC) curves, and the calculation of the area under the ROC curves (AUC). The ROC curve, plotted as the true positive fraction (TPF) against the false positive fraction (FPF) with various threshold values, is a method to analyze the diagnostic accuracy. The Area Under the ROC Curve (AUC) indicates the diagnostic accuracy. The optimal cut-off point was calculated based on the mean value plus two standard deviations of the HDs. The difference between groups was tested using the Mann–Whitney U-test to determine statistical significance; *p* < 0.05 were considered significant.

## Results

### Characterization of the mAb to mono-Ln-γ2 (2H2 mAb)

**Fig. 1:**
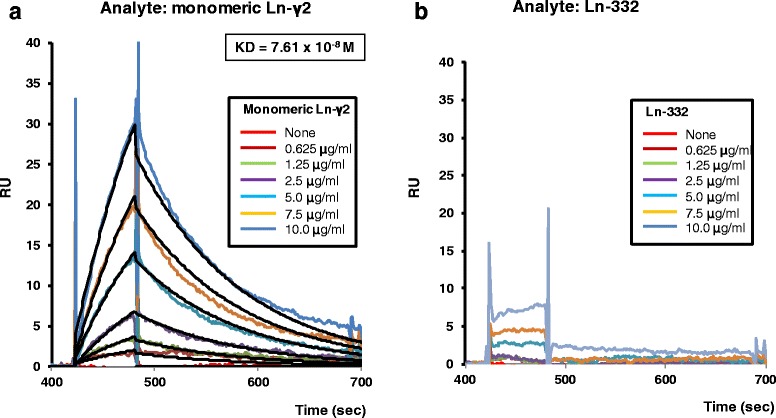
SPR analysis of the specificity of the 2H2 mAb using a BIAcore 3000. **a** Sensorgrams for the interactions between the 2H2 monoclonal antibody (mAb) and mono-Ln-γ2 and **b** between the 2H2 mAb and Ln-332. The 2H2 mAb was immobilized onto the surface of CM5 chips, varying concentrations of mono-Ln-γ2 and Ln-332 were injected, and binding was monitored using surface plasmon resonance. The injected concentrations of mono-Ln-γ2 and Ln-332 were 0, 0.625, 1.25, 2.5, 5.0, 7.5, and 10.0 μg/mL

To confirm the specific binding reactivity of 2H2 mAb to Ln-γ2 chain, we systematically measured the equilibrium dissociation constants (Kd) for the binding of the 2H2 mAb to mono-Ln-γ2 or heterotrimeric Ln-332 using SPR analysis to confirm the specificity of the 2H2 mAb (Fig. 1 and Additional file 2: Supplementary Methods). When we immobilized the 2H2 mAb on the CM5 sensor chip and tested the various concentrations of mono-Ln-γ2 and Ln-332, we found that the 2H2 mAb bound the mono-Ln-γ2 in a concentration-dependent manner, although this result was not observed for Ln-332. We also analyzed the quality of Ln-332 using SPR with an anti-Ln-γ2 mAb-conjugated sensor chip, and confirmed that it bound the chip in a concentration-dependent manner (Additional file 3: Figure S2). The Kd values were calculated using the Langmuir 1:1 model, although we did not calculate the Kd values for Ln-332, as they were below the limit of detection. These results suggest that the 2H2 mAb bound specifically to mono-Ln-γ2 but not to the γ2 chain of Ln-332.

### CLIA for mono-Ln-γ2

**Fig. 2:**
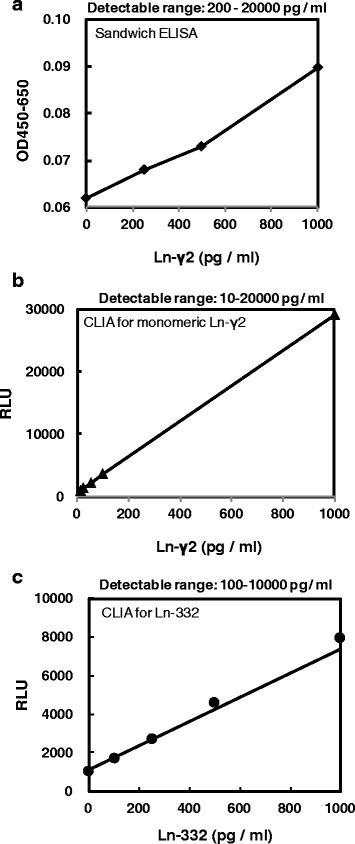
Calibration curves. **a** The standard curve from an ELISA for Ln-γ2 using a conventional Ln-γ2 antibody, the D4B5 mAb, and the DIII pAbs. The detection range was 200–20,000 pg/mL. **b** The standard curve for the chemiluminescence immunoassay (CLIA) using the 2H2 mAb and the DIII pAbs. The detection range was 10–20,000 pg/mL. The CLIA had 20-fold greater sensitivity compared with the sandwich ELISA. **c** Standard curve for Ln-332 using the CLIA with an anti-Ln-α3 mAb and the anti-DIII pAbs. The detection range was 100–10,000 pg/mL

In a previous study, a sandwich ELISA with a conventional mAb to Ln-γ2 (D4B5) was used to test for Ln-γ2 in urine, and the detection range was found to be 200–1000 pg/mL [13]. To improve this range, we used the 2H2 mAb rather than D4B5 in the CLIA. When we analyzed the diluted recombinant mono-Ln-γ2 in the buffer solution (0–20,000 pg/mL), we found that the detection range was 10–20,000 pg/mL (approximately 20-fold better than the sandwich ELISA) (Fig. 2 and Additional file 4: Figure S3). The spiked recovery rates in the three urine specimens were 82.3–90.3% for the 50 pg/mL specimens and 86.1–96.2% for the 1000 pg/mL specimens (Additional file 5: Figure S4). These ranges are acceptable for making a clinical diagnosis.

### Evaluation of Ln-γ2 in urine specimens

**Fig. 3:**
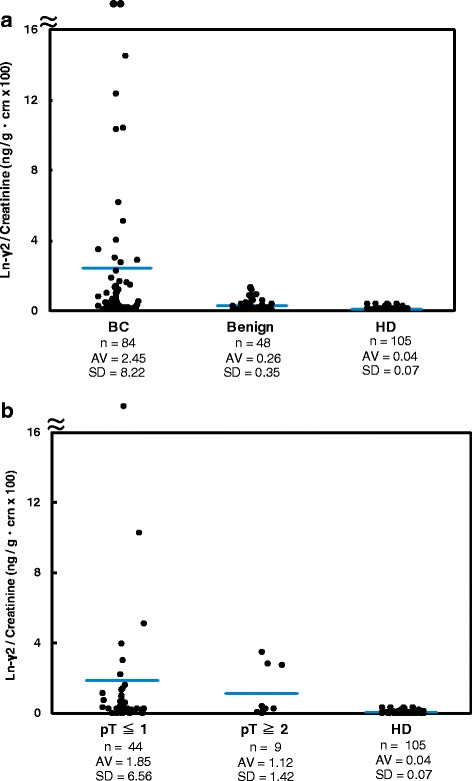
Dot plot analysis of urine mono-Ln-γ2 with BC, benign urological disease, and HD. Dot plots of creatinine-corrected urine Ln-γ2, (Ln-γ2/crn) in patients with urological diseases. **a** Dot plots of Ln-γ2/crn in 84 patients with bladder cancer (BC), 48 patients with benign disease, and 105 healthy donors (HDs). The standard deviation values for urinary Ln-γ2 were small in patients with benign diseases and HDs. The distributions of urine Ln-γ2/crn values in patients with BC were significantly higher compared with those from patients with benign disease and HDs (*p* = 0.0006 and *p* < 0.0001, respectively). **b** Dot plots of Ln-γ2/crn in 44 patients with non-muscle invasive BC (NMIBC), nine patients with muscle invasive BC (MIBC), and 105 HDs. The distributions of urine Ln-γ2/crn values in the NMIBC and MIBC groups were significantly higher than those from the healthy donors (*p* < 0.0001)

To evaluate the utility of mono-Ln-γ2 to diagnose BC, we measured the mono-Ln-γ2 values in urine specimens using CLIA. In total, 237 urine specimens were obtained from 84 patients with BC, 48 patients with benign diseases, and 105 HDs (Additional file 6: Figure S5), and standardized the values by the urine creatinine (Fig. 3a). The mean values for mono-Ln-γ2 were 2.45 ± 8.22 for BC cases, 0.26 ± 0.35 for benign disease cases, and 0.04 ± 0.07 ng/g · crn (×100) for HDs. The standard deviation values for mono-Ln-γ2 in patients with benign diseases or HDs were small and tightly gathered around the mean value. The distributions of mono-Ln-γ2 values in the BC cases were significantly broader than those from patients with benign disease and HDs (*p* = 0.0006 and *p* < 0.0001, respectively). The cut-off value for mono-Ln-γ2 was defined as 0.19 ng/g · crn (×100), based on the mean value plus two standard deviations for the HDs. Using this cut-off value, we detected 61% (51/84) of the patients with BC. Furthermore, we diagnosed 61% (27/44) of the patients with NMIBC and 67% (6/9) of the patients with MIBC (Table 1). The mono-Ln-γ2 mean values in the urine from NMIBC and MIBC cases were 1.85 ± 6.56 and 1.12 ± 1.42 ng/g · crn (×100), respectively (Fig. 3b). These distributions were noticeably different from those among HDs (*p* < 0.0001).

**Fig. 4:**
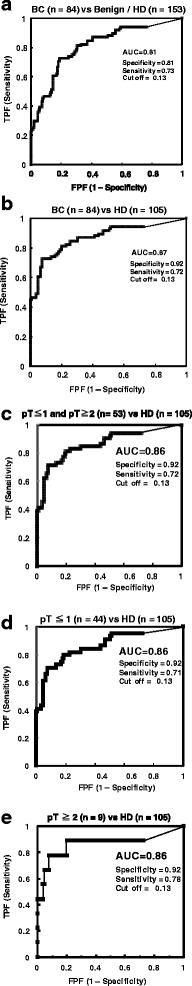
Receiver operating characteristic (ROC) curve analysis. **a** Bladder cancer (BC) vs. benign urological disease and healthy donors (HDs), (**b**) BC vs. HDs, (**c**) non-muscle invasive BC (NMIBC; ≤pT1) and muscle invasive BC (MIBC; ≥pT2) vs. HDs, (**d**) NMIBC vs. HDs, and (**e**) MIBC vs. HDs. The area under the curve (AUC) value for BC vs. benign disease and HDs (**a**) was 0.87, and the AUC value for BC vs. HDs (**b**) was 0.81. These AUC values are sufficient to facilitate a diagnosis of BC. The AUC values for NMIBC and MIBC (**c**), NMIBC (**d**), and MIBC (**e**) were all 0.86. Thus, mono-Ln-γ2 in urine can be used to diagnose both NMIBC and MIBC

To evaluate the diagnostic accuracy of mono-Ln-γ2, ROC curve analysis was performed (Fig. 4). The AUC for diagnosing BC (vs. patients with benign disease and health donors) was 0.81, and a cut-off value of 0.13 ng/g · crn (×100) provided a specificity of 0.81 and a sensitivity of 0.73. The AUC for diagnosing BC (vs. only HDs) was 0.87, and the same cut-off value provided a specificity of 0.92 and a sensitivity of 0.72. These AUC values were sufficient to facilitate a diagnosis of BC (Fig. 4a–b). The ROC analyses were also performed separately using NMIBC and MIBC cases (Fig. 4d–e). The AUC values for both NMIBC and MIBC were 0.86, and a cut-off value of 0.13 ng/g · crn (×100) provided a specificity of 0.92 and sensitivity of 0.71 (NMIBC) and 0.78 (MIBC).

**Fig. 5:**
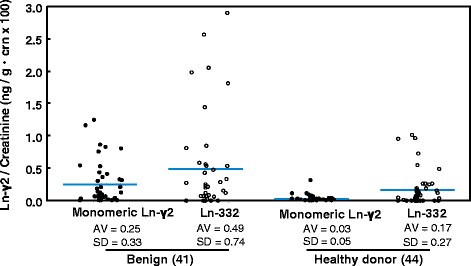
Comparison of mono-Ln-γ2 and Ln-332 values in patients with benign urological diseases and HDs. Dot plots of creatinine-corrected urine Ln-γ2 (Ln-γ2/crn) and Ln-332 (Ln-332/crn) from 41 patients with benign urological diseases and 44 healthy donors. The distributions of urine Ln-332/crn were wider than the distributions of urine Ln-γ2/crn in patients with benign urological diseases and in the healthy donors. These results indicate that Ln-332 is secreted into the urine of patients with benign urological disease and healthy donors, which could lead to false-positive diagnoses

We also analyzed the Ln-332 values from the urine of patients with benign disease and HDs using the CLIA incorporating an anti-Ln-α3 mAb (Fig. 2c). This test revealed moderate and high values of Ln-332 in the urine of patients with benign diseases and even among HDs (Fig. 5). The mono-Ln-γ2 and Ln-332 mean values from the 41 patients with benign diseases were 0.25 ± 0.33 and 0.49 ± 0.74 ng/g · crn (×100), respectively. The mono-Ln-γ2 and Ln-332 mean values from the 44 HDs were 0.03 ± 0.05 and 0.17 ± 0.27 ng/g · crn (×100), respectively. The standard deviation values were smaller for mono-Ln-γ2 in patients with benign diseases or HDs, compared with those for Ln-332. This is presumably because there might have been false-positive Ln-332 reactions in our previous sandwich ELISA, and our present results suggest that the CLIA using the 2H2 mAb detects mono-Ln-γ2 specifically.

## Discussion

We developed a CLIA detection system to improve the sensitivity and specificity of mono-Ln-γ2 to diagnose BC clinically. This CLIA system uses a general-purpose device for clinical testing, and provides faster processing, higher throughput capacity, lower background signal, and better sensitivity compared with our previous sandwich ELISA. Furthermore, the CLIA system uses a mono-Ln-γ2-specific 2H2 mAb rather than D4B5. D4B5 recognizes both forms of Ln-γ2; therefore, our previous sandwich ELISA presumably reacted with both mono-Ln-γ2 and the γ2 chain of heterotrimeric Ln-332 in urine specimens from patients with BC, patients with benign diseases, and HDs [13]. In this context, Ln-332 is a major component of the basement membrane, and previous studies have revealed that the γ2 chain of Ln-332 can be detected in serum specimens from patients with malignant and benign diseases [18–21]. In the present study, our CLIA was able to detect elevated values of Ln-332 in the urine of patients with benign urological disease (Fig. 5), which may explain the false-positive reactions. However, the 2H2 mAb reacts predominantly with mono-Ln-γ2, rather than Ln-332, as reported previously [16, 17]. After evaluating clinical urine specimens, we observed that the Ln-γ2 values in patients with benign diseases or HDs were smaller and more tightly gathered around the mean value compared with the results from our sandwich ELISA (Fig. 3a).

We also confirmed that the CLIA provides a detection range of 10–20,000 pg/mL in <30 min, which represents approximately 20-fold greater sensitivity compared with our previous sandwich ELISA. In addition, the spiked recovery test confirmed that the CLIA can detect Ln-γ2 in urine specimens, and that the recovery rate was clinically acceptable. Moreover, the Ln-γ2 values determined in patients with benign urological disease (e.g., benign prostatic hyperplasia, epididymitis, urinary stones, and urethral injury) were comparable to those detected in HDs (Fig. 3a). Based on these findings, we believe that our CLIA can recognize mono-Ln-γ2 selectively in urine specimens, even in patients with benign urological diseases.

The patients with BC had higher mono-Ln-γ2 values than patients with benign urological disease or HDs (Fig. 3b). These values were lower than the values from the sandwich ELISA. This is presumably because the CLIA is not susceptible to Ln-332 contamination in the urine specimens, as it can accurately detect only mono-Ln-γ2. Thus, we consider that the present CLIA enhances the reliability of mono-Ln-γ2 to diagnose BC.

Although urine cytology and cystoscopy are used to diagnose BC, there are no reliable biomarkers that can be used to screen for low-grade NMIBC (≤ pT1). In the present study, the ROC curve analysis revealed that mono-Ln-γ2 could be used to screen for BC, especially NMIBC (AUC = 0.86 for ≤pT1 vs. HDs) (Fig. 5). Therefore, we are preparing a larger clinical study to evaluate the use of mono-Ln-γ2 as a urine biomarker for NMIBC among Japanese patients.

## Conclusion

We developed a CLIA system to detect mono-Ln-γ2 in urine using a specific mAb. Although further analysis is needed to determine whether mono-Ln-γ2 also appears in the urine of patients with other urological cancers, such as prostate and kidney cancer, the present results indicate that this system might be useful to diagnose BC and NMIBC, especially compared with current diagnostic markers for BC.

## Additional files

Additional file 1: Supplementary Figure S1. (PDF 71 kb)

Additional file 2: Supplementary Methods and references. (PDF 68 kb)

Additional file 3: Supplementary Figure S2. (PDF 124 kb)

Additional file 4: Supplementary Figure S3. (PDF 61 kb)

Additional file 5: Supplementary Figure S4. (PDF 72 kb)

Additional file 6: Supplementary Figure S5. (PDF 77 kb)

## Abbreviations

AUC: Area under the curve
BC: Bladder cancer
BTA: Bladder tumor antigen
CLIA: Chemiluminescence immunoassay
DIII: Laminin-γ2 domain III
ELISA: Enzyme-linked immunosorbent assay
FPF: False positive fraction
HD: Healthy-donor
Ln-332: Laminin-332
mono-Ln-γ2: Monomeric laminin-γ2
NMIBC: Non-muscle invasive BC
NMP-22: Nuclear matrix protein-22
ROC: Receiver operating characteristic
SPR: Surface plasmon resonance
TPF: True positive fraction
