# Supplementary figures and images for: Development of a fully automated chemiluminescence immunoassay for urine monomeric laminin-γ2 as a promising diagnostic tool of non-muscle invasive bladder cancer
Source: Biomark Res. 2017 Oct 13;5:29. doi: 10.1186/s40364-017-0109-4 (PMC5640956; doi:10.1186/s40364-017-0109-4)

## Additional File 1

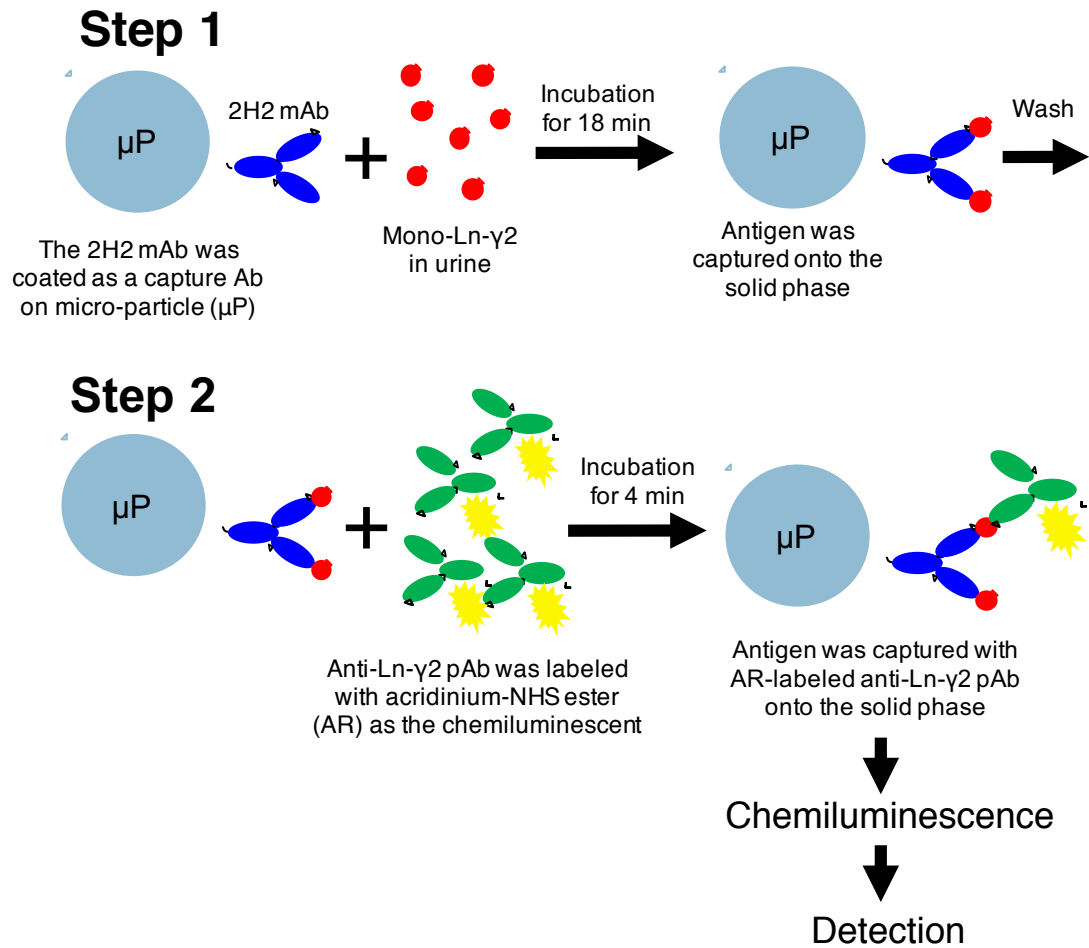

**Fig S1.**

A schematic representation for the principle of the CLIA assay

Supplement: Supplementary file 1 — Supplementary Figure S1. (PDF 71 kb) [file 40364_2017_109_MOESM1_ESM.pdf]
