# Supplementary material for: Development of a fully automated chemiluminescence immunoassay for urine monomeric laminin-γ2 as a promising diagnostic tool of non-muscle invasive bladder cancer
Source: Biomark Res. 2017 Oct 13;5:29. doi: 10.1186/s40364-017-0109-4 (PMC5640956; doi:10.1186/s40364-017-0109-4)
Supplement: Supplementary file 2 — Supplementary Methods and references. (PDF 68 kb) [file 40364_2017_109_MOESM2_ESM.pdf]

Additional file 2.

Supplementary Methods

Monoclonal antibody to monomeric Ln-γ2 (mono- Ln-γ2)

A previous report described a hybridoma cell that produces a mAb that targets mono-Ln-γ2 (clone name: 2H2 mAb), which was established at Institute of Medical Science, University of Tokyo. [1] The IgG1 protein was purified from the hybridoma serum-free culture medium using a Hi-Trap Protein-G HP column (GE Healthcare), as described previously. [1]

Preparation of recombinant mono-Ln-γ2 and Ln-332 proteins

The recombinant mono-Ln-γ2 protein was purified using spent medium from an MDCK transfectant expressing an intact Ln-γ2 protein and a 2H2 mAb-conjugated Protein A Sepharose affinity column, as previously reported. [1] Purified recombinant Ln-332 protein was purchased from Oriental Yeast (Shiga, Japan).

Supplementary Reference

1. Koshikawa, N., Minegishi, T., Nabeshima, K. et al.: Development of a new tracking tool for the human monomeric laminin-gamma 2 chain in vitro and in vivo. Cancer Res, **68:** 530, 2008
