# Supplementary material for: Development of a fully automated chemiluminescence immunoassay for urine monomeric laminin-γ2 as a promising diagnostic tool of non-muscle invasive bladder cancer
Source: Biomark Res. 2017 Oct 13;5:29. doi: 10.1186/s40364-017-0109-4 (PMC5640956; doi:10.1186/s40364-017-0109-4)
Supplement: Supplementary file 3 — Supplementary Figure S2. (PDF 124 kb) [file 40364_2017_109_MOESM3_ESM.pdf]

Additional File 3

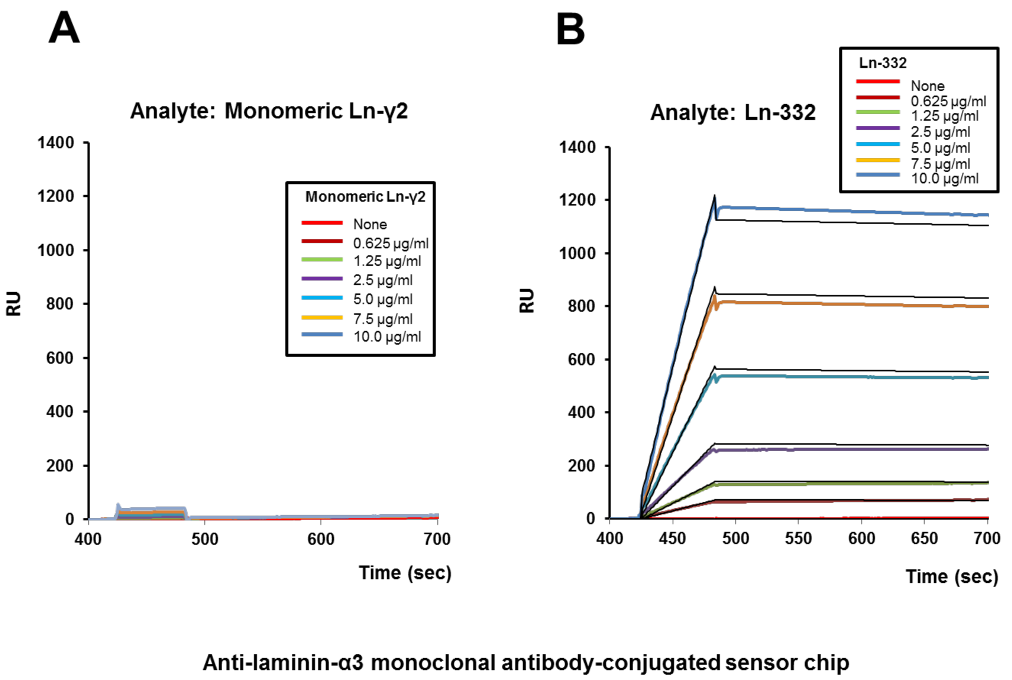

Figure S2.

The Surface-Plasmon-Resonance (SPR) analysis of 2H2 mAb specificity by a BIAcore 3000. (A) The sensorgrams of interactions between anti-Ln-α3 mAb and mono-Ln-γ2 and (B) between anti-Ln-α3 mAb and Ln-332 (B) are analyzed. The anti-Ln-α3 mAb were immobilized onto the surface of CM5 chips. Various concentrations of mono-Ln-γ2 or Ln-332 protein was injected and binding was monitored by SPR. Concentrations of mono-Ln-γ2 and Ln-332 injected were 0, 0.625, 1.25, 2.5, 5.0, 7.5 and 10.0 μg/ml. The anti-Ln-α3 mAb reacted with Ln-332 protein in dose-dependent manner.
