# Supplementary material for: Development of a fully automated chemiluminescence immunoassay for urine monomeric laminin-γ2 as a promising diagnostic tool of non-muscle invasive bladder cancer
Source: Biomark Res. 2017 Oct 13;5:29. doi: 10.1186/s40364-017-0109-4 (PMC5640956; doi:10.1186/s40364-017-0109-4)
Supplement: Supplementary file 4 — Supplementary Figure S3. (PDF 61 kb) [file 40364_2017_109_MOESM4_ESM.pdf]

Additional file 4

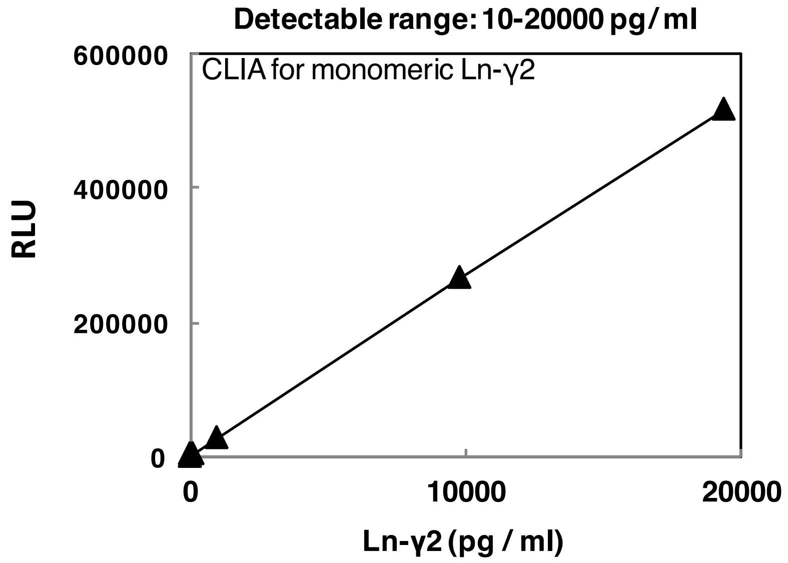

Figure S3.

The standard curve for the chemiluminescence immunoassay (CLIA) using the 2H2 mAb and the DIII pAb.
