# Supplementary material for: Development of a fully automated chemiluminescence immunoassay for urine monomeric laminin-γ2 as a promising diagnostic tool of non-muscle invasive bladder cancer
Source: Biomark Res. 2017 Oct 13;5:29. doi: 10.1186/s40364-017-0109-4 (PMC5640956; doi:10.1186/s40364-017-0109-4)
Supplement: Supplementary file 5 — Supplementary Figure S4. (PDF 72 kb) [file 40364_2017_109_MOESM5_ESM.pdf]

Additional file 5

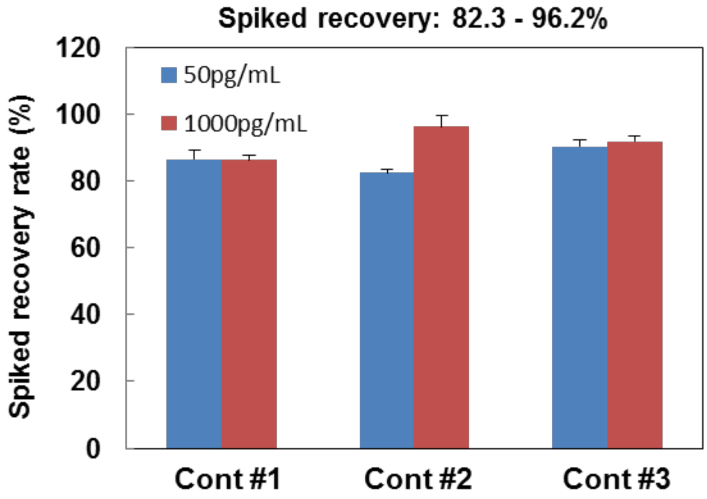

Figure S4.

Spiked recovery. The results of the spiked recovery test. Purified mono-Ln-γ2 protein was added to 3 normal urine specimens to achieve final concentrations of 50 and 1,000 pg/mL. The samples were tested using the CLIA and the recovery rate was calculated for the purified mono-Ln-γ2. The recovery rates from three healthy urine specimens were 82.3–90.3% for a final concentration of 50 pg/mL and 86.1–96.2% for a final concentration of 1,000 pg/mL.
