# Supplementary material for: Development of a fully automated chemiluminescence immunoassay for urine monomeric laminin-γ2 as a promising diagnostic tool of non-muscle invasive bladder cancer
Source: Biomark Res. 2017 Oct 13;5:29. doi: 10.1186/s40364-017-0109-4 (PMC5640956; doi:10.1186/s40364-017-0109-4)
Supplement: Supplementary file 6 — Supplementary Figure S5. (PDF 77 kb) [file 40364_2017_109_MOESM6_ESM.pdf]

Additional file 6

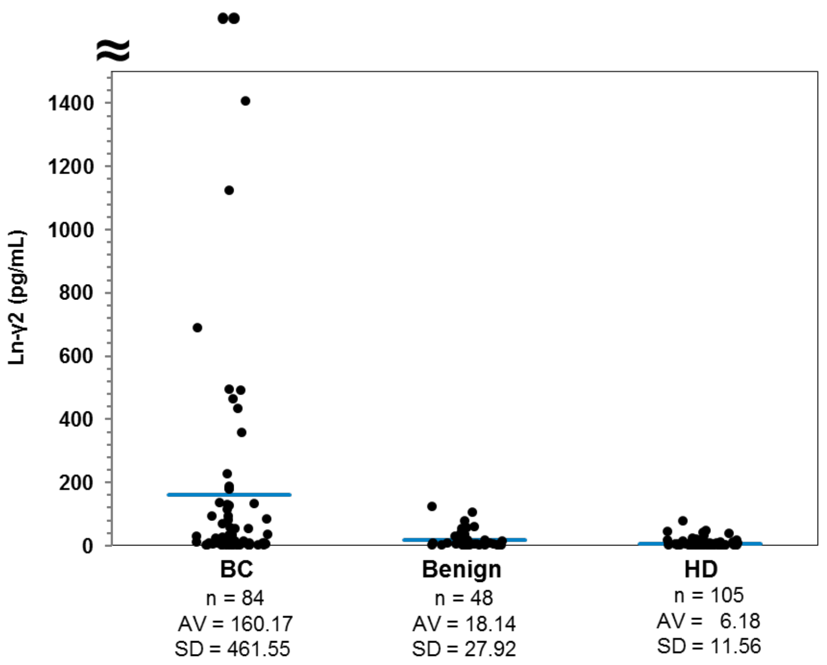

Figure S5.

Dot plot analysis of urine mono-Ln-γ2 with BC, benign urological disease, and HD without creatinine standardization. Dot plots of Ln-γ2 in 84 patients with bladder cancer (BC), 48 patients with benign disease, and 105 healthy donors (HDs). The standard deviation values for urinary Ln-γ2 were small in patients with benign diseases and HDs.
